# Methylation profiles of imprinted genes are distinct between mature ovarian teratoma, complete hydatidiform mole, and extragonadal mature teratoma

**DOI:** 10.1038/s41379-020-00668-8

**Published:** 2020-09-01

**Authors:** Noriko Kato, Akihisa Kamataki, Hidekachi Kurotaki

**Affiliations:** 1 Department of Anatomic Pathology, Hirosaki University School of Medicine and Hospital, Hirosaki, Japan; 2 Department of Anatomic Pathology, Hirosaki University Graduate School of Medicine, Hirosaki, Japan; 3 Department of Pathology, Aomori Prefectural Central Hospital, Aomori, Japan

**Keywords:** Oncogenesis, Epigenetics

## Abstract

Mature ovarian teratoma is considered to be a parthenogenetic tumor that arises from a single oocyte/ovum. Conversely, complete hydatidiform mole (CHM) is androgenetic in origin: classic CHM arises from a single or two sperm. Since mature ovarian teratoma and CHM have only maternal and paternal genomes, respectively, their genome imprinting is theoretically reverse, but this has yet to be investigated. Genome imprinting in struma ovarii, a special form of mature teratoma, remains unclear. Although a mature teratoma can rarely arise in extragonadal sites, its genome imprinting, as well as cell origin, is poorly understood. One of the most important mechanisms of genome imprinting is DNA methylation. To investigate the methylation profile of imprinted genes, we performed methylation-specific multiplex ligation-dependent probe amplification (MS-MLPA) of 21 imprinting control region (ICRs) of 9 imprinted genes/gene clusters in formalin-fixed, paraffin-embedded samples obtained from 12 mature ovarian teratomas, 6 struma ovarii, 10 CHMs, and 7 extragonadal (1 sacrococcygeal, 6 mediastinal) mature teratomas of females. In mature ovarian teratomas, ICRs of maternally and paternally imprinted genes showed high and low levels of methylation, respectively, and this pattern was almost reverse in CHMs. In CHMs, however, some ICRs showed aberrant methylation. The methylation profile of struma ovarii was comparable to that of mature ovarian teratomas, except for an adenomatous tumor. In extragonadal mature teratomas, the methylation pattern was somatic or irregular. In conclusion, mature ovarian teratomas/struma ovarii, CHMs, and extragonadal mature teratomas showed distinct methylation profiles of imprinted genes. Ovarian teratomas and CHMs are most likely to inherit their methylation profiles from their ancestral germ cells, although some aberrant methylation suggests a relaxation of imprinting in CHMs and a subset of struma ovarii. Extragonadal mature teratomas may carry a methylation profile of misplaced primordial germ cells or possibly somatic cells that have been reprogrammed in vivo.

## Introduction

Mature teratomas are composed of an array of mature tissues derived from two or three embryonic layers and are classified as a subgroup within germ cell tumors. In the ovary, mature teratomas are considered to be parthenogenetic tumors that arise from a single oocyte/ovum showing meiotic failure [1–3]. In contrast, complete hydatidiform mole (CHM), a representative pregnancy-related disorder, is androgenetic in origin: classic CHMs arise from a single or two sperm [4–6]. From the standpoint of parent-of-origin, ovarian teratomas and CHMs are very similar to the phenotypes of mouse embryos obtained by nuclear transplantation: parthenogenetic (bi-maternal) embryos develop well but show the poor development of trophoblasts, whereas androgenetic (bi-paternal) conceptuses fail to develop but show marked proliferation of trophoblasts [7–9]. These findings indicate that the maternal and paternal genomes are different regarding the expression of genes, and they carry imprints in a parent-of-origin manner. In humans, an estimated 100–200 imprinted genes have been identified [10]. The parent-specific expression of imprinted genes is regulated by different epigenetic mechanisms, especially by DNA methylation [11].

Mature ovarian teratomas and CHMs are expected to show an opposite methylation profile of imprinted genes, since they have only maternal and paternal genomes, respectively. However, this has not yet been studied systemically. Mature ovarian teratomas rarely show a predominant or exclusive proliferation of thyroid tissue, designated as struma ovarii [12]. It remains unclear if mature ovarian teratomas and struma ovarii have a common methylation profile of imprinted genes. Mature teratomas arise not only in the gonads but also at extragonadal midline sites, including the sacrococcyx and mediastinum. There are limited data available on genome imprinting in extragonadal teratomas.

In the present study, we analyzed a series of mature ovarian teratomas, CHMs, struma ovarii, and extragonadal mature teratomas of females to compare their methylation profiles of imprinted genes using a methylation-specific multiplex ligation-dependent probe amplification (MS-MLPA) method.

## Materials and methods

### Materials

Twelve cases of mature ovarian teratoma, ten cases of CHM, six cases of struma ovarii, and seven cases of extragonadal mature teratomas of females (one sacrococcygeal, six mediastinal) were retrieved from the pathology archive between 2010 and 2019. None of the mature teratomas were associated with any immature elements or other germ cell tumor components. Struma ovarii was defined as a tumor that contained thyroid tissue comprising at least >80% of tumor areas. All CHMs were confirmed by genotyping as well as p57^KIP2^ immunohistochemistry (Supplementary). All the mature teratomas and struma ovarii had been surgically resected. All but one CHM had been curetted, while one case of CHM had involved emergency hysterectomy at the 15th week of gestation because of pregnancy-induced hypertension. None of the CHMs had developed invasive mole or choriocarcinoma. All materials had been fixed in 10% formalin and embedded in paraffin. This study was approved by the Institutional Ethics Committee (approval code: 2018-1158, Hirosaki University Graduate School of Medicine).

### DNA extraction

Formalin-fixed, paraffin-embedded (FFPE) blocks containing representative lesions were selected from each case. FFPE blocks containing the following somatic tissue were used as controls: fallopian tube or contralateral normal ovary (for ovarian teratomas and struma ovarii), decidua or endometrium (for CHMs), and thymus or peritumoral connective tissue (for extragonadal teratomas).

Serial 10-um-thick FFPE sections were used for total DNA isolation. Areas of each section containing lesion or control tissue were manually dissected using a sterile scalpel. In mature teratomas and struma ovarii, dense inflammatory infiltrate within the tumor was carefully excluded to avoid contamination by somatic cells. Genomic DNA was extracted from the dissected tissue with the QIAamp DNA FFPE Tissue Kit (QIAGEN, Hilden, Germany), according to the manufacturer’s instructions.

### Methylation-specific multiplex ligation-dependent probe amplification (MS-MLPA)

For methylation analysis, the methylation-specific SALSA MS-MLPA Kit ME034-B1 (MRC-Holland, Amsterdam, The Netherlands) was used according to the manufacturer’s instructions. It was shown that MS-MLPA could be applied successfully to DNA derived from FFPE [13]. MS-MLPA using ME034-B1 facilitated the simultaneous characterization of a total of 21 imprinting control regions (ICRs) of the following 9 imprinted genes or gene clusters: *H19*, *MEG3*, *GNAS* cluster (*NESP55*, *NESPAS*, *GNASXL*, and *GNAS*), *PLAGL1*, *GRB10*, *MEST*, *KCNQ1OT1*, *SNRPN*, and *PEG3*. *H19*, *MEG3*, and *NESP55* are paternally imprinted genes, whereas *PLAGL1*, *GRB10*, *MEST*, *KCNQ1OT1*, *SNRPN*, *PEG3*, *NESPAS*, *GNASXL*, and *GNAS* are maternally imprinted genes (Supplementary). Two hundred fifty nanogram of DNA was used in each MS-MLPA reaction. All reactions were performed in a Verti 96 Well Thermal Cycler (Applied Biosystems) in triplicate. Fragment separation was done by capillary electrophoresis on an Applied Biosystems 3500 Genetic Analyzer. Peak patterns were evaluated using Coffalyser.Net (v.140721.1958, MRC-Holland).

In somatic cells, the average methylation level of ICRs is expected to be 50%, since somatic cells have one paternal and one maternal allele. In cells of parthenogenetic origin, the average methylation level of ICRs is theoretically 100 and 0% in maternally and paternally imprinted genes, respectively, since they have only maternal alleles. This is the reverse in cells of androgenetic origin.

## Results

### Clinicopathological features

Patients’ age, including the estimated gestational age (for CHM cases), and tumor size of mature teratomas and struma ovarii are summarized in Table 1. The mean age was significantly older in patients with struma ovarii (54.3 years) than those with mature ovarian teratomas (28.9 years) and mediastinal teratomas (25 years). Three patients with mature ovarian teratomas, none with struma ovarii, and three patients with extragonadal teratomas were aged between 0 and 18 years. There was no difference in tumor size among mature ovarian teratomas, struma ovarii, and extragonadal teratomas.

**Table 1:** Summary of cases.

Histology	*n*	Age (years)	Tumor size (cm)
		(mean)	(mean)
Mature ovarian teratoma	12	1–48	4–12
		(28.9)	(7.8)
Struma ovarii			
Typical	5	37–77	4–7
Resembling follicular adenoma	1	46 (54.3)	7 (6)
Complete hydatidiform mole	10	18–49^a^ (33.1)	–
Extragonadal teratoma of female			
Sacrococcygeal	1	3 days^b^	3.5
Mediastinal	6	13–38 (25)	5–15 (8.1)

^a^Gestational age was 7–15 (mean: 10.1) weeks.

^b^This tumor was congenital, and it was resected 3 days after birth.

**Fig. 1:**
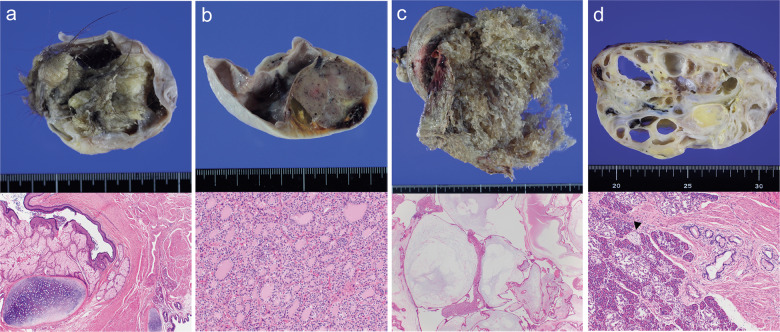
Pathological features of mature ovarian teratoma, struma ovarii, complete hydatidiform mole, and mediastinal mature teratoma. (**a**) Mature ovarian teratoma is a grossly cystic tumor with hairs and sebaceous material. On histology, skin with pilosebaceous units, cartilage, smooth muscle, and respiratory epithelium are identified (corresponding to case 7 of Fig. 2). (**b**) In struma ovarii, the gross section shows a brownish nodular area, associated with a cystic area. Histologically, the nodular area resembles a thyroid adenoma with mixed normofollicular and microfollicular pattern (corresponding to case 2 of Fig. 2). (**c**) In complete hydatidiform mole, molar tissue has a grossly “bunch of grapes” appearance, filling the uterine cavity. Histologically, hydropic villi with central acellular cisternae, trophoblastic inclusions, and circumferential trophoblastic proliferation are evident (corresponding to case 10 of Fig. 2). (**d**) Mediastinal mature teratoma shows a cystic and solid cut surface. On histology, various tissues, including pancreatic tissue, intestinal mucosa, smooth muscle, and nerve tissue (arrowhead), are identified (corresponding to case 2 of Fig. 2).

Representative pathological features are shown in Fig. 1. All 12 mature ovarian teratomas contained tissues of 2 or 3 germ layers, including skin, neural tissue, adipose tissue, bone, cartilage, and bronchial or gastrointestinal mucosa. Among the six struma ovarii, four were exclusively composed of thyroid tissue, and the other two had a minor component of mature teratoma. The former included a nodular lesion resembling follicular adenoma, which had not metastasized or recurred for 6 years after surgery. One sacrococcygeal teratoma and two mediastinal teratomas contained pancreatic tissue as well as intestinal mucosa. All CHMs showed hydropic villi with central acellular cisternae, trophoblastic inclusions, and circumferential trophoblastic proliferation.

### Methylation status of imprinted genes

**Fig. 2:**
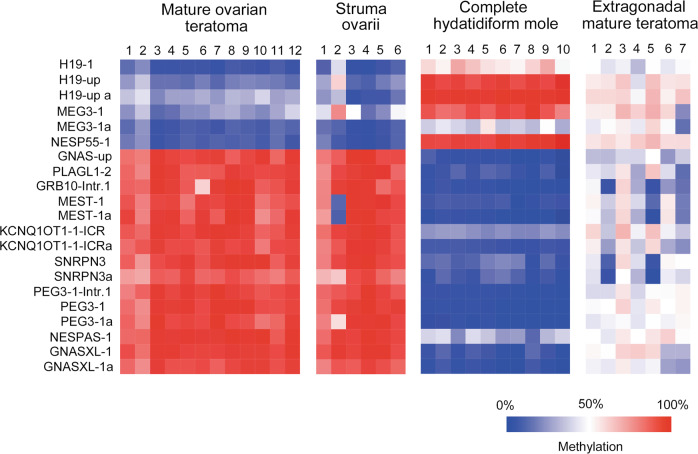
Methylation status of imprinting control regions (ICRs) of imprinted genes/gene clusters in mature ovarian teratomas, struma ovarii, complete hydatidiform mole, and extragonadal mature teratomas of females. Numbers indicate cases in order of age. The color gradient from blue–white–red represents low to high percentages (0–50–100%) of the average methylation level. In mature teratomas and struma ovarii, the methylation levels of ICRs of maternally and paternally imprinted genes were high and low, respectively, although one struma ovarii resembling follicular adenoma (case 2) showed aberrant methylation levels in some ICRs. In CHMs, the overall methylation pattern was opposite to that observed in mature ovarian teratomas and struma ovarii. However, some ICRs of *MEG3*, *H19*, and *NESPAS* showed aberrant methylation levels. Extragonadal mature teratomas showed somatic or irregular methylation patterns (case 1, sacrococcygeal teratoma; cases 2–7, mediastinal teratomas).

The average methylation level of a total of 21 ICRs is shown in Fig. 2. In mature ovarian teratomas, ICRs of maternally imprinted genes showed high methylation levels, whereas ICRs of paternally imprinted genes showed low methylation levels. In CHMs, the overall methylation pattern was opposite to that observed in mature ovarian teratomas, but aberrant methylation was noted in some ICRs, including those of *MEG3*, *H19*, and *NESPAS*. In struma ovarii, the methylation pattern was comparable to that observed in mature ovarian teratomas, although one struma ovarii resembling follicular adenoma (case 2) showed aberrant methylation in some ICRs. Extragonadal mature teratomas showed somatic or irregular methylation patterns.

## Discussion

In the present study, three histologic groups: mature ovarian teratomas/struma ovarii, CHMs, and extragonadal mature teratomas, showed distinct methylation patterns of a series of imprinted genes.

Mature ovarian teratomas are considered to be parthenogenetic tumors that arise from a single oocyte that has fallen into meiotic failure or a single ovum that has undergone endoreduplication [1–3]. In either case, mature ovarian teratomas have only maternal genomes. Concordantly, the average methylation level of maternally and paternally imprinted genes was high and low, respectively, in all 12 cases examined. A previous study focusing on five imprinted genes generated similar data in pediatric cases [14]. Recently, genome-wide methylation analysis revealed that mature ovarian teratomas show a general trend toward maternal imprinting [15]. During germ cell development in females, imprinting takes place in oocytes of the fetal ovary at a late stage of gestation, and it is almost completed soon after birth [15]. In the present study, the age of patients with mature ovarian teratomas ranged from 1 to 48 years, but there was no significant difference in the methylation pattern depending on patients’ age. It is suggested that the imprinting is maintained by oocytes throughout their life and mature ovarian teratomas inherit it from their ancestral oocytes or ova.

Struma ovarii is defined as a special form of mature teratoma composed predominantly or exclusively of thyroid tissue [12]. Actually, thyroid tissue is a common component of a mature teratoma: up to 20% of mature teratomas may contain thyroid tissue [16]. In the present study, the overall methylation pattern in struma ovarii was comparable to that in mature ovarian teratomas. Our previous study showed that the genetic zygosity of struma ovarii is concordant with that of mature teratomas [17]. These data suggest that struma ovarii and mature teratomas share a pathogenetic basis, although the reason why some mature teratomas develop struma ovarii and others do not remains unclear. In struma ovarii, thyroid tissue appears normal or rarely resembles follicular neoplasm [16]. In our series, one of the six cases was a nodular lesion resembling follicular adenoma. Intriguingly, this case showed aberrant methylation in some ICRs, whereas the others did not. There is a possibility that maintenance of differential methylation of imprinted genes alters during adenomatous proliferation in struma ovarii.

The androgenetic origin of CHMs was first shown by Kajii and Ohama [4]. Approximately 80% of CHMs arise from duplication of the haploid genome of a single sperm, whereas the other 20% are considered to arise from dispermy, the fertilization of the egg by two sperm [5]. In either case, androgenetic CHMs have only the paternal genome. Concordantly, the overall methylation level of maternally and paternally imprinted genes was low and high, respectively, in all 10 CHMs examined. However, methylation levels of some imprinted genes deviated from this pattern. In particular, an ICR of *MEG3*, a paternally imprinted gene, was significantly hypomethylated. *MEG3* composes the imprinted gene cluster *DLK1/DIOS3* on 14q32.2, and encodes long noncoding RNA [18]. Recent accumulating data demonstrate that noncoding RNAs are not the result of transcriptional noise, but play an important role in biological functions, such as placental and embryonic growth and pluripotency maintenance [18]. In CHMs, the expression and function of *MEG3* noncoding RNA remain unclear. Other imprinted genes, including *H19* and *NESPAS*, also showed slightly aberrant methylation. As for *H19*, previous studies reported an unexpected expression of *H19* noncoding RNA in intervillous trophoblasts of CHMs, and proposed the concept “relaxation of imprinting” [19, 20]. Relaxation of imprinting is also likely to explain an unanticipated expression of p57^KIP2^ in CHMs: *p57*^*KIP2*^ is a paternally imprinted gene that should not be expressed in CHMs, but is expressed in intervillous trophoblasts, which paradoxically serves as an internal positive control of p57^KIP2^ immunohistochemistry [21]. It has long been hypothesized that relaxation of imprinting is relevant to the malignant potential of CHMs: CHMs are associated with a ten times greater risk of progressing to choriocarcinoma/persistent trophoblastic disease than partial hydatidiform moles [6]. Further study is needed to clarify if aberrant methylation of imprinted genes is responsible for the relaxation of imprinting and malignant potential of CHMs.

Mature teratomas arise primarily within the gonads, but a minority can also arise at extragonadal midline sites, including the sacrococcyx and mediastinum [22]. It has been suggested that they arise from primordial germ cells (PGCs) that migrate along the midline toward the gonads in an early embryonic stage. In the present study, all extragonadal teratomas of females, including three pediatric cases, did not show a maternal methylation pattern, which is characteristic of ovarian teratomas. This finding may support a recent perspective that pediatric germ cell tumors are a developmental disease: failures of PGC specification, migration, and proliferation, as well as failures of egg/sperm differentiation, are implicated in susceptibility to germ cell tumors [23]. Sacrococcygeal teratomas are most commonly seen as congenital neoplasms [24]. According to previous studies, sacrococcygeal teratomas showed a bimodal global methylation and somatic imprinting status [15]. This profile is comparable with that of early PGCs, which are on the global demethylation and prior to imprinting erasure [15]. In the present study, one sacrococcygeal teratoma of a neonate showed a nearly somatic methylation pattern of imprinted genes, consistent with previous data. On the other hand, there is little information about DNA methylation or the imprinting status in mediastinal teratomas [25]. In the present study, six mediastinal teratomas showed somatic or irregular methylation patterns of imprinted genes. During embryogenesis, the mediastinum is outside the route of PGC migration. Misplaced PGCs have been considered to be an origin of mediastinal teratomas, but they have never been identified. Recently, a study using in vivo reprograming mice showed that teratomas develop in a variety of organs, including the kidney, pancreas, intestine, and adipose tissue [26]. There is a possibility that in vivo reprogramming occurs in somatic cells of humans, and that such reprogrammed cells are origins of extragonadal teratomas. There is little information available about the methylation profile of animal teratomas obtained by in vivo reprograming. It is an open question whether human teratomas can originate from a reprogrammed somatic cell at extragonadal sites.

In summary, methylation profiles of imprinted genes were distinct between mature ovarian teratomas/struma ovarii, CHMs, and extra-ovarian teratomas. Mature ovarian teratomas showed high and low methylation of maternally and paternally imprinted genes, respectively, being concordant with their parthenogenetic origin. This pattern was recapitulated by struma ovarii, except for the adenomatous form. In CHMs, the overall methylation pattern was opposite to that observed in mature ovarian teratomas, but some imprinted genes showed aberrant methylation, suggesting a relaxation of imprinting. In sacrococcygeal and mediastinal mature teratomas of females, the methylation pattern was somatic or irregular. From the current concept of reprogramming in vivo, there is a possibility that extragonadal teratomas originate from a reprogrammed somatic cell, as well as a PGC. Future studies along with experimental models will shed light on the enigmatic origin of extragonadal teratomas.

## Supplementary information

Supplementary
