# Supplementary material for: Methylation profiles of imprinted genes are distinct between mature ovarian teratoma, complete hydatidiform mole, and extragonadal mature teratoma
Source: Mod Pathol. 2020 Sep 1;34(2):502–7. doi: 10.1038/s41379-020-00668-8 (PMC7817522; doi:10.1038/s41379-020-00668-8)
Supplement: Supplementary file 1 — Supplementary [file 41379_2020_668_MOESM1_ESM.pdf]

Short tandem repeat (STR) genotyping

For diagnosis of complete hydatidiform mole (CHM), genotyping was performed by multiplex polymerase chain reaction (PCR) at 6 STR loci. The microsatellite DNA markers used were: D6S2439, D6S1056, D9S1118, D4S2639, D17S1290, and D20S480. These 6 markers were used because of their high discriminating power in the Japanese population.[1] Details of the methods have been described in our previous study.[2] Cases were classified as CHM when STR results showed complete nonidentity (no matching alleles) between villous and maternal DNAs at 2 or more loci, according to the criteria of Furtado et al.[3]

In all 10 cases examined, the STR data showed complete nonidentity between villous and maternal DNAs at 2-6 loci and fulfilled the criteria for CHM (Supplementary Fig.1).

Immunohistochemistry for $p57^{Kip2}$

For complementary diagnosis of CHM, immunohistochemistry for $p57^{Kip2}$ was performed using FFPE sections. The sections were subjected to Leica BOND III

(Leica Biosystems, Newcastle, UK). The primary antibody used was a rabbit monoclonal antibody against human $p57^{Kip2}$ (clone EP2515Y; dilution 1:200; Abcam, Cambridge, UK). The immunoreaction was visualized using a BOND Polymer Refine Detection kit (Leica Biosystems). Distinct nuclear staining for $p57^{Kip2}$ was evaluated as positive.

In all 10 cases examined, $p57^{Kip2}$ immunostaining was not detected in villous cytotrophoblasts or stromal cells, although immunostaining was retained in intervillous trophoblasts (Supplementary Fig.2).

References

1. Ohtaki H, Yamamoto T, Yoshimoto T, Uchini R, Ooshima C, Katsumata Y, et al. A powerful, novel, multiplex typing system for six short tandem repeat loci and the allele frequency distributions in two Japanese regional populations. Electrophoresis 2002; 23: 3332-3340.

2. Kato N, Sakamoto K, Murakami K, Iwasaki Y, Kamataki A, Kurose A. Genetic zygosity of mature ovarian teratomas, struma ovarii, and ovarian carcinoids. Virchows Archiv 2018; 473: 177-182.

3. Furtado LV, Paxton CN, Jama MA, Tripp SR, Wilson AR, Lyon E, et al. Diagnostic utility of microsatellite genotyping for molar pregnancy testing. Arch Pathol Lab Med 2013; 137: 55-63.

Figure legends

Figure 1 Representative genotyping results for complete hydatidiform mole. Each locus in the villi is homozygous, and the allele in the villi is not present in the mother. Complete homozygosity across all 6 STR loci is consistent with monospermy.

Figure 2 Immunohistochemistry for $p57^{Kip2}$ in complete hydatidiform mole. Villous cytotrophoblasts and stromal cells were negative for $p57^{Kip2}$, although intervillous trophoblasts (arrowheads) were positive for it.

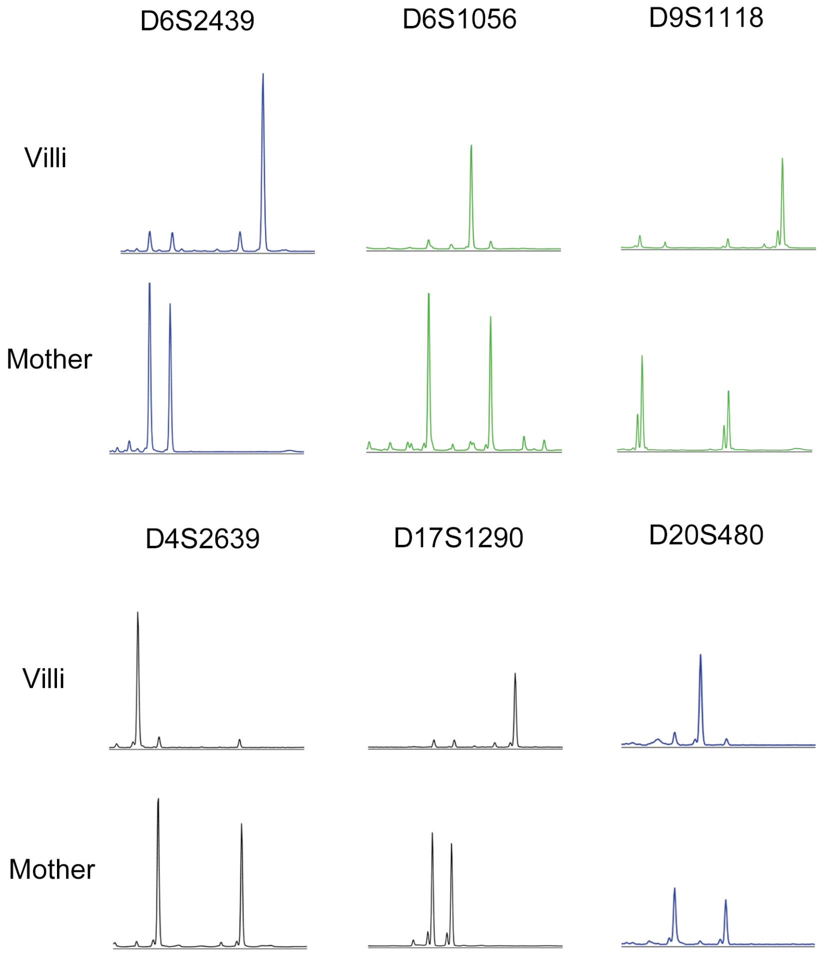

Supplementary Fig.1

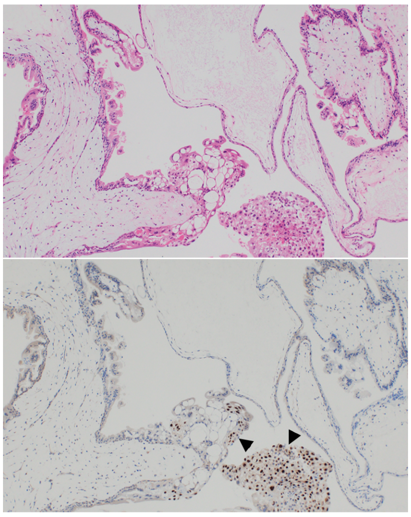

Supplementary Fig.2

Supplementary

Table List of 21 probes for methylation-specific multiplex ligation-dependent probe amplification (MS-MLPA)

Probe	Gene/Gene cluster (Chromosome)	Imprinted allele	Probe target information*
	***H19* (11p15.5)**		
H19-1	Exon 1	Paternal	11-001.975955
H19-up	Upstream	Paternal	11-001.976269
H19-up a	Upstream	Paternal	11-001.976583
	***MEG3* (14q32.2)**		
MEG3-1	MEG3_TSS_DMR	Paternal	14-100.361799
MEG3-1a	MEG3_TSS_DMR	Paternal	14-100.362079
	***PLAGL1* (6q24.2)**		
PLAGL1-2	PLAGL1_TSS_alt-DMR	Maternal	06-144.370600
	***GRB10* (7p12.2)**		
GRB10-Intr.1	Intron 1	Maternal	07-050.817995
	***MEST* (7q32.2)**		
MEST-1	MEST_alt-TSS _DMR	Maternal	07-129.918586
MEST-1a	Exon 1	Maternal	07-129.919370
	***KCNQ1OT1* (11p15.5)**		
KCNQ1OT1-1-ICR	Exon 1	Maternal	11-002.677118
KCNQ1OT1-1-ICRa	Exon 1	Maternal	11-002.677593
	***SNRPN* (15q11.2)**		
SNRPN3	Exon 5 (CpG island)	Maternal	15-022.751214
SNRPN3a	Exon 5 (CpG island)	Maternal	15-022.751773
	***PEG3* (19q13.43)**		
PEG3-1-Intr.1	Intron1	Maternal	19-062.041776
PEG3-1	Exon 1	Maternal	19-062.043503
PEG3-1a	Exon1	Maternal	19-062.044027
	***GNAS* cluster (20q13.32)**		
NESP55-1	NESP55-Exon 1	Paternal	20-056.848321

GNAS-up	GNAS Upstream	Maternal	20-056.897762
NESPAS 1	NESPAS Exon 1	Maternal	20-056.859408
GNASXL-1	GNASXL Exon 1	Maternal	20-056.863512
GNASXL-1a	GNASXL Exon 1	Maternal	20-056.863588

* https://genome-asia.ucsc.edu/cgi-bin.hgGateway?db=hg18&redirect=manual&source=genome.ucsc.edu
